# The potential modal shift and health benefits of implementing a public bicycle share program in Montreal, Canada

**DOI:** 10.1186/1479-5868-10-66

**Published:** 2013-05-24

**Authors:** Daniel Fuller, Lise Gauvin, Yan Kestens, Patrick Morency, Louis Drouin

**Affiliations:** 1 Department of Community Health and Epidemiology, University of Saskatchewan, Health Sciences Building, 107 Wiggins Road, Saskatoon, SK S7N 5E5, Canada; 2 Centre de recherche du Centre Hospitalier de l’Université de Montréal, C.P. 6128, Succursale Centre-ville, Montréal, QC, H3C 3J7, Canada; 3 Département de médecine sociale et préventive, Université de Montréal, C.P. 6128, Succursale Centre-ville, Montréal, QC, H3C 3J7, Canada; 4 Direction de santé publique de Montréal, Université de Montréal, 1301, rue Sherbrooke Est, Montréal, QUE, H2L 1M3, Canada; 5 Département de médecine sociale et préventive, Université de Montréal, 1301, rue Sherbrooke Est, Montréal, QUE, H2L 1M3, Canada

**Keywords:** Bicycle share program, Intervention, Life days gained, Modal shift, Transportation, Public transportation, Modal integration

## Abstract

**Background:**

This study estimated the modal shift associated with the implementation of a public bicycle share program in Montreal, Canada.

**Methods:**

A population-based sample of adults participated in two cross sectional telephone surveys. Self-reported travel behaviors were collected at the end of the first (fall 2009) and second (fall 2010) season of implementation. The sample included 2502 (Mean age=47.8 years, 61.8% female), and 2509 (Mean age=48.9 years, 59.0% female) adult respondents in each survey.

**Results:**

The estimated modal shift associated with the implementation of the PBSP from motor vehicle use to walking, cycling, and public transportation was 6483 and 8023 trips in 2009 and 2010. This change represents 0.34% and 0.43% of all motor vehicle trips in Montreal.

**Conclusions:**

The implementation of a PBSP was associated with a shift toward active transportation. The modal shift was complex and not simply the result of a discrete shift from one mode to another. Promotion of active transportation should encourage integration of multiple active transportation modes to better reflect people’s actual transportation behaviors.

## 

The relationship between transportation and health is of growing interest in public health [1,2]. Transportation systems can have health impacts [3] and the literature shows that many of these health impacts are associated with roads and motor vehicles [4]. Numerous studies show that greater use of motor vehicles at the population level is associated with negative health consequences including injuries and death due to collisions [5,6], exposure to air pollution [7], and lower levels of physical activity [8]. These health consequences are of primary concern for public health. One method of reducing these negative health consequences is to reduce motor vehicle use by promoting active modes of transportation (i.e., public transportation, walking, and cycling). Trips of less than 5 kilometres are particularly amenable to active transportation because they are competitive in terms of time and cost compared to motor vehicles [9]. Replacing short motor vehicle trips by active transportation would contribute to health via the daily accumulation of health enhancing physical activity [10,11]. Multiple studies show associations between a high prevalence of active transportation and lower traffic related injuries, [12] and greater physical activity [10,11]. Modelling studies suggest that despite the fact that pedestrians and cyclists are at greater risk of injury from collisions with motor vehicle users [13] the overall benefits in terms of cardiovascular health of increasing active transportation outweigh the risks [14]. Public transportation is also the safest mode of transportation [13].

Public health interventions that promote a modal shift from motor vehicle use to active forms of transportation (i.e., walking, cycling, public transportation) can simultaneously influence multiple health outcomes, including physical activity, injuries and exposure to air pollution [15]. A small number of studies to date have evaluated the effect of transportation interventions on modal shift [16,17], and researchers have predominantly relied on modelling studies that often make overly optimistic assumptions about intervention effects [18-20]. Public bicycle share programs (PBSP) are one intervention that has the potential to create a modal shift. PBSPs increase population access to cycling by making bicycles available at docking stations throughout an area within a city for a fee [21,22]. For example, Montreal’s BIXI (BIcycle-taXI) program, North America’s largest in 2012, makes available 5050 bicycles at 405 docking stations within an area with a population density of approximately 12,000 residents/km^2^[23]. The PBSP is implemented in areas with good access to public transportation in the form of subway and bus service [24]. PBSPs are understudied. There are also a number of challenges when examining the contribution of different transportation modes to total transportation [22,25,26]. Modal shift and multi-modal transportation are overlapping concepts and interventions with the potential to increase modal shift could have unintended health and mobility consequences.

The aim of this study was to estimate the modal shift from motor vehicles to active transportation associated with the implementation of the PBSP program in Montreal. An additional aim was to estimate the modal shift relative to the total number of motor vehicle trips per day on the Island of Montreal.

## Methods

### Design

Data from two cross sectional studies were used. Two population-based samples of adults participated in telephone surveys conducted at the end of the first season of the PBSP implementation (October 8^th^ - December 12^th^ 2009) and at the end of the second season of implementation (November 8^th^ - December 12^th^ 2010). The PBSP in Montreal is removed during the winter.

The sampling frame for each survey was individuals residing on the Island of Montreal with a landline telephone. Within contacted households the available individual to next celebrate a birthday and aged 18 years or older was invited to respond. To recruit a sufficient number of participants exposed to the PBSP intervention, the sampling frame was divided into two strata defined by the presence or absence of PBSP docking stations in the neighbourhood of residence (neighbourhood refers to City of Montreal neighbourhood boundaries). In the first strata, where PBSP docking stations were not available, random digit dialling to landlines was used to contact those residing on the Island of Montreal. In the second strata, where PBSP docking stations were available, oversampling was conducted by randomly selecting landlines with Montreal postal codes matched to neighbourhoods were PBSP docking stations were available.

### Procedures

Ethical approval was obtained from the *Centre de Recherche du Centre Hospitalier de l’Université de Montréal*. Respondents were recruited via a polling firm with verbal informed consent obtained prior to participation. Responses could be given in French or English. The research team trained telephone interviewers from the polling firm and performed on going quality surveillance to ensure the survey was being conducted in accordance with training.

### Measures

The survey included health, transportation and opinion measures, which were used to evaluate the impact of the PBSP on cycling in Montreal [25,27]. Four variables were used to estimate the total modal shift from motor vehicles to active transportation for the population of Montreal; modal shift, multi-modal transportation, cycling as primary mode of transportation, and new trips. For 2009 or 2010, PBSP user modal shift was measured by asking “what was the mode of transportation you used to make the trips that you now make with BIXI bicycles?” Responses were personal bicycle, walking, public transportation, taxi, and car. Multiple responses were not possible. The multi-modal transportation question asked PBSP users “do you integrate other modes of transportation into your travel when you use BIXI bicycles?” Responses included taking the bus, subway, taxi or walking at the beginning or end of a PBSP trip. Multiple responses were possible.

Modal shift for non-PBSP users was measured by asking “even if you do not use BIXI bicycles yourself, has the availability of BIXI made you change your habitual modes of transportation?” Possible responses included cycling, walking, taking public transportation, using taxis more, and driving less. Multiple responses were possible. We assume that because the PBSP was a large scale intervention on the built environment in Montreal that it could plausibly promote a shift toward active modes of transportation for non PBSP users.

For both PBSP users and non-users, new trips were measured by asking “has the availability of BIXI bicycles encouraged you to make trips that you would not have made otherwise?” Responses included to work or school, for leisure or fun, for exercise, for shopping, for social visits to family and friends, trips for work and no more trips. Multiple response categories were possible.

The questions used to examine modal shift did not specifically ask respondents to estimate how many trips per day were replaced by the implementation of the PBSP, rather these were general questions about modal shift. In order to estimate modal shift we assumed that each respondent made 2.3 trips per day, the average number of trips in Montreal based on data from the 2008 Montreal Household Travel Survey [28]. Additionally, we assumed that the fraction of trips per person per day being replaced or integrated was 5% (0.115 trips/day), 10% (0.23 trips/day), or 15% (0.345 trips/day) of the total 2.3 trips per person per day. We believe that these assumptions are realistic and sufficiently conservative. We could have assumed that 100% of trips were replaced or integrated as a result of the PBSP but this assumption is not realistic and would over estimate the modal shift.

### Data analysis

Population prevalence and 95% confidence intervals (95% CI) were estimated for the modal shift, multi-modal transportation, and new trips. Estimates were computed for the 2009 and 2010 surveys. Inverse probability of selection and post stratification weighting was applied to all analyses. For respondents reporting use of the PBSP, population prevalence estimates were computed for modal shift from all modes to PBSP use and multi-modal trips that integrated more than one mode during PBSP trips. For non-PBSP users population prevalence estimates were computed for modal shift from any one mode to any other mode. For the entire sample, including PBSP and non-PBSP users, the total number of new trips was estimated.

Equation 1 shows the formula used to calculate the modal shift.

(1) $$\begin{array}{ll} \left[\left(\mathrm{ModalShif}t_{\mathrm{User}}\right)\right) & \left(-(\mathrm{Cyclis}t_{\mathrm{User}}+\mathrm{MultiMode}l_{\mathrm{User}})\right)\\ & \left(+\mathrm{ModalShif}t_{\mathrm{Nonuser}}\right]-\mathrm{NewTrip}s_{\mathrm{Population}} \end{array}$$

The modal shift from motor vehicles to active transportation associated with the implementation of the PBSP in Montreal was estimated by adding modal shift for PBSP users and non-users and subtracting multi-modal trips, and cycling as a primary mode of transportation from users, and subtracting new trips for both users and non-users. Multi-modal trips were subtracted because respondents reporting modal shift and multi-modal trips of the same mode would have been counted twice. For example, if a respondent reported shifting from public transportation to the PBSP and integrating public transportation and PBSP we did not want to double count those responses. Cycling as a primary mode of transportation was subtracted because a shift from cycling to PBSP use does not represent a shift in mode. Modal shift estimates were computed for 2009 and 2010 and compared to data from Montreal’s most recent travel survey in 2008 [28].

## Results

The sample included 2502 (Mean age=47.8 years, 61.8% female), and 2509 (Mean age=48.9 years, 59.0% female) adult respondents in each survey. The response rates were 34.6% and 35.7%, respectively for each survey. Socio-demographic and weighting results have been published elsewhere [29].

Estimates show that approximately 124,094 (8.10%) and 168,665 (11.01%) individuals residing on the Island of Montreal used the PBSP at least once in 2009 and 2010, respectively. There was a significant increase in PBSP use between season 1 and season 2 (F=6.97, *p*=0.001).

Table 1 shows the estimated number of PBSP users reporting a modal shift. The results show that the majority of PBSP users shifted from using public transportation (n=62,558; 50.43%) in 2009 and (n=69,008; 40.91%) in 2010. Related to motor vehicle transportation, 7.92% and 10.13% of PBSP users reported replacing motor vehicle use with PBSP trips in 2009 and 2010 respectively. At the population level this 10.13% in 2010 translates to 17,078 unique users, which based on our assumption of 0.115 trips (5%) replaced represents 1964 fewer automobile trips per day.

**Table 1:** Modal shift prevalence of people using the PBSP in Montreal in 2009 and 2010

	**2009**	**2010**
	**Estimated Number (Prevalence; 95% CI)**	**Estimated Number (Prevalence, 95% CI)**
Estimated Modal Shift		
From Personal Bicycle	29,290.93 (23.61%; 16.44, 30.78)	36,691.22 (21.75%; 15.43, 28.08)
From Walking	22,373.49 (18.04%; 11.82, 24.25)	36,109.02 (21.41%; 15.00, 27.81)
From Public Transit	62,558.32 (50.43%; 40.59, 60.28)	69,008.04 (40.91%; 33.49, 48.34)
From Taxi	--	9,778.66 (5.80%; 2.23, 9.37)
From Motor Vehicle	9,818.83 (7.92%; 3.49, 12.34)	17,077.61 (10.13%; 6.03, 14.22)
PBSP Users	124,094.43 (8.10%; 6.71, 9.74)	168,664.55 (11.01%; 9.46, 12.56)

Table 2 shows the results from population prevalence estimates of modal shift for non PBSP users. Non PBSP users reported more use of active modes of transportation and less motor vehicle use. An estimated 148,983 (10.58%) of non users reported more walking, cycling, or public transportation use while 11,429 (0.81%) reported less motor vehicle use in 2009. The 2010 estimates were similar to those in 2009 with 143,784 (10.55%) of non users reporting more walking, cycling or public transportation use while 8,435 (0.62%) reported less motor vehicle use. Estimates for the total number of new trips generated from implementation of the PBSP were 12,982 and 11,166 in 2009 and 2010, respectively assuming a modal shift of 5% of trips per day.

**Table 2:** Modal shift prevalence of people not using the PBSP in Montreal in 2009 and 2010

	**2009**	**2010**
	**Estimated number (Prevalence, 95% CI)**	**Estimated number (Prevalence; 95% CI)**
No change	1246225 (88.51%, 86.83; 90.19)	1211146 (88.83%, 87.15, 90.52)
More cycling	66320 (4.71%, 3.55; 5.87)	65609 (4.81%, 3.64; 5.98)
More walking	41084 (2.92%, 2.09; 3.75)	38432 (2.82%, 1.92; 3.72)
More public transportation	41579 (2.95%, 2.01; 3.90)	39743 (2.92%, 1.99, 3.84)
More taxi	1352 (0.10%, -0.04; 0.23)	1309 (0.10%, -0.04; 0.23)
Less car	11429 (0.81%, 0.43; 1.19)	8435 (0.62%, 0.33; 0.91)
Non PBSP Users	1407936 (91.90%, 90.26; 93.29)	1363353 (88.99%; 87.34; 90.45)

Note. Based on adult population of 1532030 minus the estimated population of PBSP users of 124,094 in 2009 and of 168,665 in 2010 residing on the Island of Montreal.

Table 3 shows that in 2009 and 2010, 91.2% and 91.3%, respectively, of respondents did not report an increase in trips as a result of the bicycle share program. The most common reason for new trips was for fun, 2.8% in 2009 and 2.7% in 2010.

**Table 3:** New trips generated as a result of the PBSP in Montreal in 2009 and 2010

	**2009**	**2010**
	**Estimated Number (Prevalence; 95% CI)**	**Estimated Number (Prevalence, 95% CI)**
No new trips	1397,011.74 (91.19%; 89.74, 92.63)	1399,385.16 (91.34%; 89.90, 92.79)
To work/school	34,072.04 (2.22%; 1.36, 3.09)	26,342.03 (1.72%; 1.16, 2.27)
Fun	42,643.29 (2.78%; 1.99, 3.58)	41,065.91 (2.68%; 1.77, 3.59)
Exercise	9,324.70 (0.61%; 0.28, 0.93)	5,536.45 (0.36%; 0.13, 0.60)
Social	14,601.17 (0.95%; 0.56, 1.34)	13,970.58 (0.91%; 0.51, 1.31)
For work	12,245.36 (0.80%; 0.41, 1.19)	10,184.02 (0.66%; 0.30, 1.03)

Note. Based on adult population of 1532030 residing on the Island of Montreal.

Table 4 shows the estimated modal shift toward active transportation associated with the PBSP in Montreal assuming a 5, 10 and 15% replacement of total trips. In the 5% scenario, 6483 trips per day in 2009 and 8023 trips per day in 2010 were replaced by active transportation. Of these trips 862 (13.3% of modal shift) and 1534 (19% of modal shift) were attributable to PBSP users in 2009 and 2010. Using data from Montreal’s travel survey the modal shift assuming 5% of trips per day where replaced is estimated to be 0.34% and 0.43% of all daily motor vehicle trips in 2009 and 2010, respectively.

**Table 4:** **Estimated modal shift in 2009 and 2010 associated with bicycle share in Montreal**, **Canada**

	**5% of trips/day**		**10% of trips/day**		**15% of trips/day**	
	**2009**	**2010**	**2009**	**2010**	**2009**	**2010**
Modal shift (n)	6483	8023	12937	16046	19449	24069
PBSP user modal shift (n)	862	1534	1695	3067	2587	4601
Total motor vehicle trips (n)	1882771	1882771	1882771	1882771	1882771	1882771
Percent modal shift (%)	0.34	0.43	0.69	0.85	1.03	1.28

## Discussion

This study examined self-reported transportation behaviours of users and non-users of a PBSP in Montreal. Consistent with a previous analysis, we show increases in the number of respondents reporting use of the PBSP in the first two years of implementation [25]. The majority of PBSP users shifted from other active modes of transportation particularly public transportation toward PBSP use. PBSP users also tended to integrate multiple active modes of transportation in a single trip to meet their daily transportation needs. The population of non-PBSP users contributed to the overall modal shift. The relative modal shift of non-PBSP users was smaller than that of PBSP users. However, in absolute terms the contribution of non-users to the overall modal shift was similar to that of PBSP users.

To date there is limited evidence examining transportation modal shifts and multi-modal trips associated with actual programs and the majority of research has relied on modeling studies [17,20,30,31]. For example, Lovelace et al. propose a best case scenario in Sheffield, UK of an increase from approximately 3.5 to 11 million trips by bicycle over a 10 year period using an “integrated pro-cycling strategy” including PBSPs and many other interventions into a single “pro-cycling” intervention [17]. This assumed increase in trips is large and is an overly optimistic representation of the impact of cycling interventions and policies [32]. We observed that the percent modal shift compared to all motor vehicle trips was approximately 0.3-0.4% but hesitate to suggest whether this is a large or small effect because PBSPs are not explicitly designed to shift people directly from motor vehicle use to active transportation. Given that limited data exist examining modal shifts associated with real programs and difficulty comparing with modeling studies we cannot say whether the estimated effect in the current study is commensurate or not with other investigations. To correctly evaluate the potential of PBSPs to change overall transportation patterns other transportation interventions designed to increase active transportation or reduce motor vehicle use should be compared to results from studies examining PBSPs.

The concept of modal shift may over simplify complex travel behaviors. Travel behavior surveys suggest that trip chaining using multiple transportation modes is very common, particularly in large urban centers. The results of the current study show that PBSP users integrated walking, public transportation, and cycling and were unlikely to make strict ‘shifts’ from one mode to another. Transportation engineers suggest that PBSPs are most advantageous for short trips in densely populated areas [22]. Public health researchers suggest that short but active trips are the most amenable to change and can influence multiple health outcomes including increasing physical activity and reducing injuries due to collisions [18,26]. Public health practitioner’s promoting active transportation should encourage people to integrate multiple active modes of transportation rather than strictly use walking or cycling as this approach would better reflect people’s actual transportation behaviors.

### Limitations

Limitations include the operationalization of multi-modal transportation assumptions about individual and trip data, response bias due to self-report and not considering trip distance. The questions used in the survey did not fully capture multi-modal trips. Responses for individuals could have been counted multiple times. This bias was limited by excluding respondents reporting a modal shift from personal bicycle to PBSP use and those reporting both a modal shift and multi-modal trip using the same mode. Individual and not trip data were used for the analysis. It was assumed that a reported shift from one transportation mode to another represented 0.115 trips per day (about 5% of trips). We believe this assumption was sufficiently conservative to be justified. Self-report data may be subject to a number of biases [33]. Social desirability bias is of particular concern because respondents, particularly non PBSP users, may overestimate their shift toward active modes of transportation. As a result for non PBSP users may not be attributable to the public bicycle share program and could be attributed factors other than the bicycle share program. The modal shift estimates did not consider trip distance. Excluding long trips where walking and cycling may not be feasible would increase the public health relevance of the estimates.

## Conclusion

Results showed that the modal shift associated with the PBSP intervention was complex but changes were relatively small. PBSP users tended to shift from other active modes of transportation including public transportation, their own bicycle, and walking. The modal shift was also accompanied by increased integration of multiple modes of active transportation in a single trip. Promotion of active transportation should encourage integration of multiple active modes of transportation to better reflect people’s actual transportation behaviors.

## Abbreviations

PBSP: Public Bicycle Share Program; BIXI: BIcycle-taXI.

## Competing interests

The authors of this article do not disclose any competing interest regarding funding sources for consultancies, studies of products or any other possible conflict.

## Authors’ contributions

All authors conceptualized and designed the study. DF conducted the majority of data analysis and writing. LG, YK and PM conducted substantial review for content and clarity. All authors read and approved the final manuscript.
